# Why Does Animal-Based Food Loss and Waste Matter?

**DOI:** 10.1093/af/vfaa039

**Published:** 2020-10-30

**Authors:** Brian Lipinski

**Affiliations:** World Resources Institute, Food Program, Washington, DC

**Keywords:** food loss, food waste, measurement

**Implications**

- Food loss and waste is an increasingly important issue for companies and governments to address, with implications for the economy, the environment, and general human well-being.
- Although the amount of animal-based food that is lost or wasted is low compared with other commodities, the higher impact associated with producing animal-based food is still worth addressing.
- Additionally, COVID-19 may be causing higher levels of food loss and waste in animal-based food industries.
- Companies within the food industry can address food loss and waste through measurement and action in their own supply chains, partnership with other businesses and through creative re-uses of food that would otherwise go to waste.

According to a landmark 2011 study by the United Nations Food and Agriculture Organization (**FAO**), about one-third of all the food produced in the world is lost or wasted at some point in the food supply chain (FAO, 2011). Although the specific amount of food loss and waste varies based on commodity type, geographic location, and stage of the supply chain, this level of inefficiency has significant impacts on the economy, human well-being, and the environment. Therefore, food loss and waste reduction should be as an opportunity to save money and improve efficiency within businesses, redirect food to those who need it, and reduce environmental impacts.

## What Is Food Loss and Waste and Why Does It Matter?

Food loss and waste, defined most simply, refers to food and/or associated inedible parts that are removed from the food supply chain (Food Loss and Waste Protocol, 2016). (Some sources make a distinction between “food loss,” which occurs in the early parts of the supply chain, and “food waste” which occurs at the retail and household stages of the food supply chain. For this article, the author does not make a distinction between food loss and food waste and instead uses the phrase “food loss and waste” to encompass the entire food supply chain.) The impacts of that loss and waste are significant. According to FAO estimates, this level of inefficiency results in approximately US$940 billion per year in economic losses. It also results in significant environmental impacts. For example, food loss and waste is responsible for 8% of the world’s greenhouse gas emissions (FAO, 2015). In fact, if food loss and waste were contained to one country, that country would be the world’s third-largest emitter after the United States and China.

**Figure 1.**
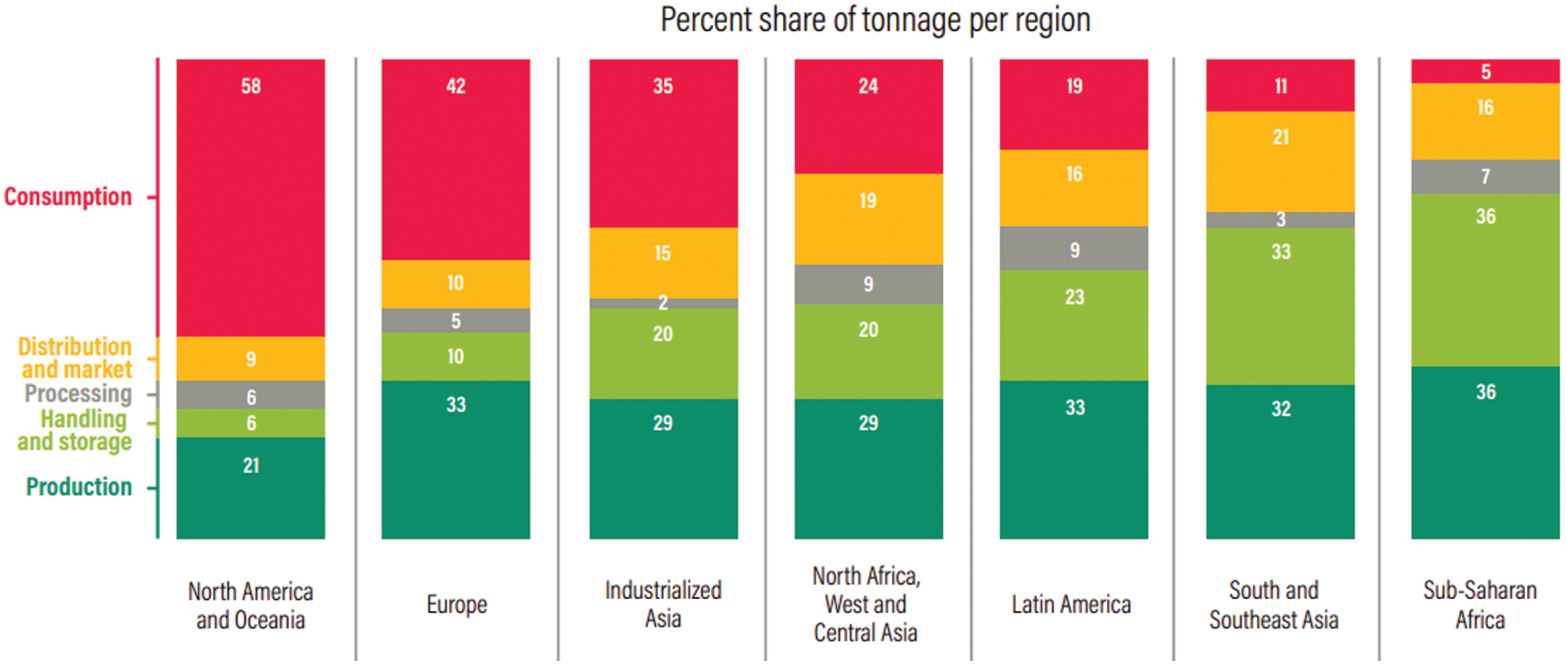
Distribution of food loss or waste by region and stage in food supply chain, 2007. *Source*: Flanagan et al. (2019).

Although food loss and waste occur across the entire food supply chain, the extent and details of where that loss and waste occur vary by region and economy. Figure 1 shows the percentage of food loss and waste occurring in each stage of the food supply chain for seven key regions. In more developed regions such as North America, Industrialized Asia and Europe, most of the loss and waste occurs toward the end of the food supply chain, at the retail and consumer levels. Conversely, in developing regions, most of the loss and waste occurs at the production and storage levels. Therefore, although food loss and waste is a significant problem across all countries, the solutions for addressing it differ greatly depending on the region and circumstances where it is occurring.

**Figure 2.**
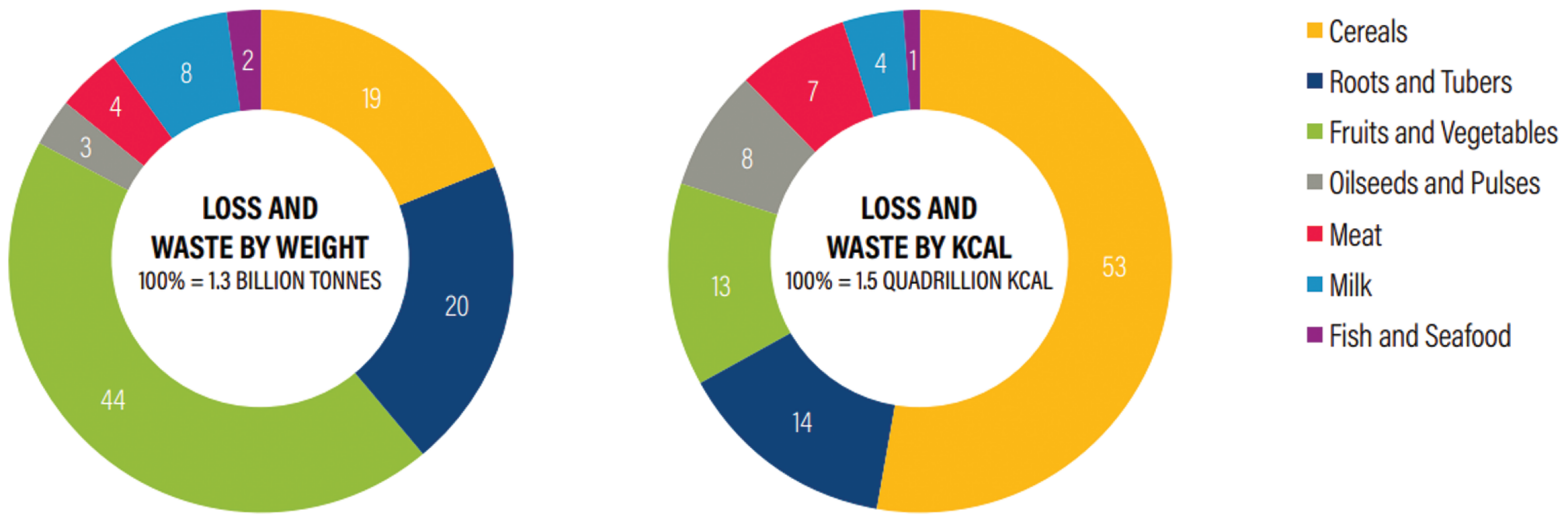
Share of global food loss and waste by commodity, 2007. *Source*: Flanagan et al. (2019).

The extent of food loss and waste also varies greatly across different types of commodities, as seen in Figure 2. When examined by weight, fruits, and vegetables make up the largest share of global food loss and waste, due in large part to the high amount of water content. When examined by caloric content, cereals and grains make up the largest share due to their high-energy density compared with other food groups.

## How Much Animal-Based Food Is Lost or Wasted?

Compared to other commodities, animal-based foods (e.g., meat, milk/dairy, and fish/seafood) are lost or wasted to a much smaller degree. As in Figure 2, animal-based foods only comprise about 12% of global food loss and waste. Typically, animal-based foods have a higher economic value than other commodity types. This may lead to this lower rate of loss and waste since the economic effect of that loss or waste is more significant than it can be with other commodity types. The COVID-19 pandemic may change this trend, however, due to unforeseen consequences within the animal-based food industry. Although comprehensive data on how COVID-19 have affected food loss and waste will likely not be available until 2021, there are numerous reports of increased food loss and waste due to the closure of meat processing facilities as well as due to the closure of restaurants and other facilities where the food would otherwise be sold (Corkery and Yaffe-Bellany, 2020).

Although animal-based products have historically been lost or wasted less than other commodities, the effects of that lost and waste are more significant than with other types of food (University of Missouri-Columbia, 2015). One reason, as mentioned above, is the higher economic value of animal-based products. Another reason is the significantly higher level of environmental inputs required to produce animal-based products. For example, when it comes to food-related greenhouse gas emissions, meat has the highest level of emissions per kilogram of food, followed by dairy (Ranganathan et al., 2016). Reducing or preventing the loss or waste of animal-related foods, therefore, has a higher impact than other commodities and should be seen as an opportunity to achieve greater economic gains while reducing environmental costs.

## What Are the Causes of Animal-Based Food Loss and Waste?

There is no single cause of animal-based food loss and waste. Food is lost and wasted throughout the entire supply chain, due to a wide variety of causes and drivers. One frequent cause of loss or waste in the animal-based food industry is spoilage during storage or transport. Since most animal-based foods require to be kept at cold temperatures, any disruption of a cold storage environment can lead to spoilage. Another cause unique to the animal-based food industry is animal sickness, which can spread quickly through livestock and lead to unnecessary animal deaths, resulting in great loss. A third such cause is discoloration of meat that can occur not necessarily due to spoilage but merely due to oxidation. Because consumers heavily rely on surface meat color when making a purchase, small spots of browning can cause consumers to reject even slightly discolored meat, which leads to waste at the retail level.

On the consumer side of the supply chain, animal-based food loss and waste tends to be due to mishandling at the household level. This can be due to improper storage (such as not keeping animal-based products at a significantly cold temperature) or failure to freeze food before it spoils. The other significant cause at the household level is insufficient knowledge about how to prepare food, which can lead to food being burnt or inedible if cooked incorrectly. Additionally, if consumers are not experienced preparing a meal in their own kitchen, they may unknowingly discard edible portions of food.

An additional cause of animal-based food loss and waste at the consumer end of the supply chain is due to misunderstandings of the meaning of food date labels. Food date labels appear in numerous forms (e.g., “best before,” “sell by,” and “use by”) and may refer to either food quality or food safety, leading to confusion for consumers. Additionally, in a recent survey, more than half of participants incorrectly believed date labeling to be federally regulated, when in fact the dates are set by manufacturers for the vast majority of products (Neff et al., 2019).

## What Is Being Done to Address Food Loss and Waste by Governments?

Food loss and waste is being addressed by governments around the world. At the international level, the most significant step has been the adoption of 17 sustainable development goals (**SDG**s) by the United Nations General Assembly, with SDG 12 seeking to “ensure sustainable consumption and production patterns.” More specifically, the third target under this goal (Target 12.3) calls for halving per capita global food waste at the retail and consumer levels and reducing food losses along production and supply chains (including postharvest losses) by 2030.

This target has focused attention on the issue of food loss and waste reduction and led to numerous initiatives and actions. The group Champions 12.3 (named for SDG Target 12.3) is one such initiative and describes itself as “a unique coalition of executives from governments, businesses, international organizations, research institutions, and civil society dedicated to inspiring ambition, mobilizing action, and accelerating progress toward achieving SDG Target 12.3.” Members include the CEOs of such prominent global food companies as Nestle, Unilever, Sodexo, and Olam International. Among other activities, Champions 12.3 publishes an annual report showing the progress the world has made toward achieving SDG 12.3 while also detailing what steps still need to be taken (Champions 12.3, 2019).

National-level strategies have also been effective in addressing food loss and waste. For example, in December 2018, the FAO launched a project to help Albania, Armenia, the Republic of North Macedonia, and the Republic of Moldova develop strategies for food loss and waste reduction (FAO, 2018). The project will also develop a regional awareness-raising campaign, establish and coordinate a partnership network, and conduct training programs in postharvest handling practices and food and waste reduction. Uganda, which experiences the highest amount of loss near the production side of the food supply chain, also has begun developing a national strategy to tackle postharvest losses in grains. The strategy will aim to increase general awareness, enhance postharvest management skills, and increase the availability of postharvest loss reducing technologies.

Countries—such as the United States, United Kingdom, The Netherlands, and Indonesia—have also found success in forming national-level partnerships to fight food waste. This has been a successful approach because it establishes relationships between sectors and entities that often do not work together and allows them to work creatively toward a common goal. The private sector members generally share best practices and innovations across the entire food supply chain, while the more prominent public figures keep food loss and waste on the national agenda by sharing success stories with media outlets and discussing the issue at events and in the media.

Many countries are also enacting legislation to incentivize food loss and waste reduction. For example, in April 2019, Argentina’s National Plan for the Reduction of Food Losses and Waste became law. The plan includes a provision through which businesses that redistribute surplus food are protected from prosecution if someone who eats the food becomes ill, as long as the business has respected relevant laws regarding food safety and expiration dates. The new law also encourages businesses to donate food products that are near to their expiration date. In July 2019, the Food Safety and Standards Authority of India passed a series of regulations to encourage individuals and organizations to donate food. The new regulations, which come into force in 2020, would protect those who donate food in good faith.

## What Are Food Companies Doing to Reduce Food Loss and Waste?

Companies at all stages of the food supply chain have begun to realize the economic benefits of reducing food loss and waste. For most companies, the journey to food loss and waste reduction begins with measurement to understand the extent and causes of food loss and waste within their operations. The data obtained through measurement then allows for targeted interventions in the company’s operation to address the most significant sources of the wastage. Then, companies continue to measure and report that data over time to understand their own progress and impacts.

One example comes from the company Nestlé, which published a detailed case study about dairy losses across farms in 30 countries (Charad et al., 2019). Nestlé conducted a multi-year measurement study to understand how much milk was being lost during transportation to their factories. They found that across the 30 countries, between 0% and 4.3% of milk was being lost during transportation. Although relatively small, the amount of loss totaled up to over 10,000 tonnes of milk loss across the 30 countries. As a result, the company undertook a series of reforms to reduce these losses, including decreasing spillage, improving training and handling procedures, and applying a strict cutoff time between milking, chilling, and processing. These measures reduced the milk waste by about one-third and generated $2.6 million in savings for farmers and Nestlé.

In addition to individual action, companies have begun to work together to address food loss and waste collectively. Companies from multiple sectors have formed partnerships and initiatives within their sector to encourage collective action among companies that might otherwise be competitors. For example, the Sustainable Rice Platform, a global multi-stakeholder alliance of some of the world’s largest rice producers and buyers, called upon its members last year to commit to halving postharvest rice losses by 2030. The Courtauld Commitment, a voluntary agreement among members of the UK retail sector, has reduced food and associated packaging waste by several million tonnes since its first phase in 2005. This sort of commitment by animal-based food businesses would be a powerful step toward reducing the impacts associated with food loss and waste by the industry.

Food companies have also taken steps to address downstream causes of food loss and waste as well. For example, in 2017, grocery manufacturers and retailers within the United States announced a voluntary initiative to simplify date labels to attempt to reduce consumer confusion and reduce associated food loss and waste (Food Industry Association, 2017). The Board of Directors for The Consumer Goods Forum, a network of 400 of the biggest consumer goods companies across 70 countries, adopted a similar initiative that same year (Consumer Goods Forum, 2017).

## How Can Companies, Farmers, and Others Get Started with Addressing Food Loss and Waste?

The most effective approach to addressing food loss and waste is the “Target-Measure-Act” approach, which leads users through three steps:

(1) *Target*: Targets set ambition, and ambition motivates action. By setting a strong target (such as the 50% reduction by 2030 target), a business sends a clear message about its intentions and has a tangible goal to work toward.
(2) *Measure*: As seen in the Nestlé example, measurement is a powerful tool for businesses that are beginning to address food loss and waste. By understanding the extent and causes of food loss and waste within a set of circumstances, plans can then be tailored to specifically address the root causes that emerge from the measurement activity. Guidance on measurement of food loss and waste can be found in the *Food Loss and Waste Accounting and Reporting Standard*, available at www.flwprotocol.org.
(3) *Act*: What ultimately matters is action. Companies, farmers, governments, and actors across all food types and all stages of the food supply chain have a part to play in food loss and waste reduction. Although the needed actions differ greatly across different commodities and sectors, the Champions 12.3 annual progress report, mentioned earlier, is an excellent resource to learn about different types of action that have been occurring to reduce food loss and waste.

## Conclusion

Although addressing food loss and waste can seem like a daunting task, the benefits are numerous. Reducing the amount of wasted food leads to economic savings, improved food availability, and reduced environmental impacts. These benefits are even larger in the animal-based food sector than for other foods, due to the higher cost and higher impacts associated with animal-based food. With numerous governments and food companies acting to reduce food loss and waste already, the issue of food loss and waste is likely to only grow in stature in the coming years.

*Conflict of interest statement*. None declared.

## About the Author

During his time with World Resources Institute, Brian Lipinski has worked extensively on the topic of food loss and waste, having served as the lead author on the paper “Reducing Food Loss and Waste” and the recent guide “How and Why to Measure Food Loss and Waste.” He is also a coauthor of the Food Loss and Waste Accounting and Reporting Standard, the world’s first global standard of requirements and guidance for quantifying and reporting on food loss and waste. He holds a Master of Science degree from the University of Michigan School of Natural Resources.
